# Pseudoangiomatous stromal hyperplasia: a rare cause of gynecomastia in men

**DOI:** 10.1080/23320885.2024.2303993

**Published:** 2024-01-18

**Authors:** Sarah Saoud, Doha Arreyouchi, Anas Ankiz, Anass Haloui, Nassira Karich, Amal Bennani, Ayat Allah Oufkir

**Affiliations:** a Department of Burns and reconstructive Surgery, Mohamed 6 hospital, Oujda Research Laboratory in Medical Sciences, Faculty of Medicine and Pharmacy of Oujda, Mohamed First University, Oujda, Morocco; b Department of Pathology, Central Laboratory, Mohammed VI University Hospital, Faculty of Medicine and Pharmacy, University Mohammed Premier, Oujda, Morocco

**Keywords:** PASH, gynecomastia, male breast

## Abstract

A 17-year-old male with chest malformation and left breast enlargement underwent surgery for gynecomastia. Histological examination revealed mammary fibrous stroma with ductal hyperplasia and features of pseudoangiomatous stromal hyperplasia. Postoperative follow-up showed no complications, but 8 months later, the patient experienced a mild recurrence with enlargement of the nipple-areolar complex. Although recommended for secondary glandular resection, the patient declined further surgery.

## Introduction

Pseudoangiomatous stromal hyperplasia (PASH) is a benign proliferative lesion of the breast stroma first described in 1986 by Vuitch and al. who identified it in nine female patients with nodular mass lesions that morphologically simulated vascular proliferative lesions [1]. Histologically, PASH is described as a dense, collagenous proliferation of mammary stroma, forming interanastomosing capillary-like spaces. The importance of this lesion is its distinction from low-grade angiosarcoma [1]. Clinically, it has a broad spectrum, ranging from an incidental microscopic finding to a palpable mass. PASH is thought to be hormonally responsive and it is typically seen in premenopausal and perimenopausal women. It is believed to be a rare finding in men [2], and is usually associated with gynecomastia [3].

## Case report

**Figure 1.**
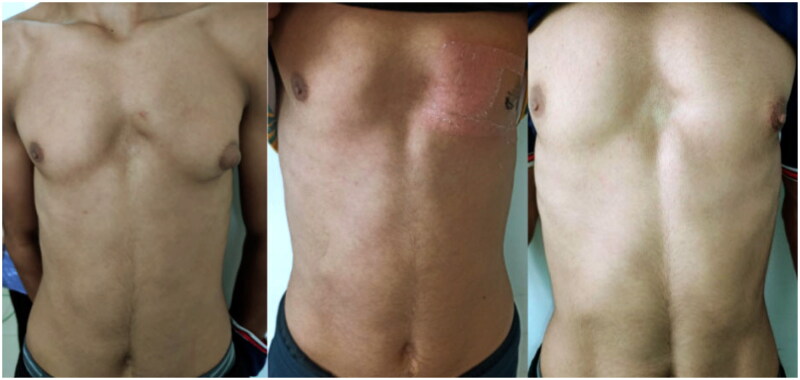
*From left to right:* preoperative image, immediate postoperative, recurrence.

We report in this paper the case of a 17-year-old man, with no past remarkable medical history except a chest malformation, and no history of medication intake. The patient reports the progressive enlargement of the left breast 2 years prior to consultation. The clinical examination found an asymmetrical breast associated with pectus excavatum which worsens the aspect of the left gynecomastia, Palpation revealed a palpable, tender, firm, mobile, disc-like mound of tissues beneath the areolar region (Figure 1). A mammary ultrasound confirmed the glandular origin of the mass. A bunch of hormonal testings were done that came normal; a testicular examination and ultrasound were performed to assess for signs of testicular tumor, which was negative.

**Figure 2.**
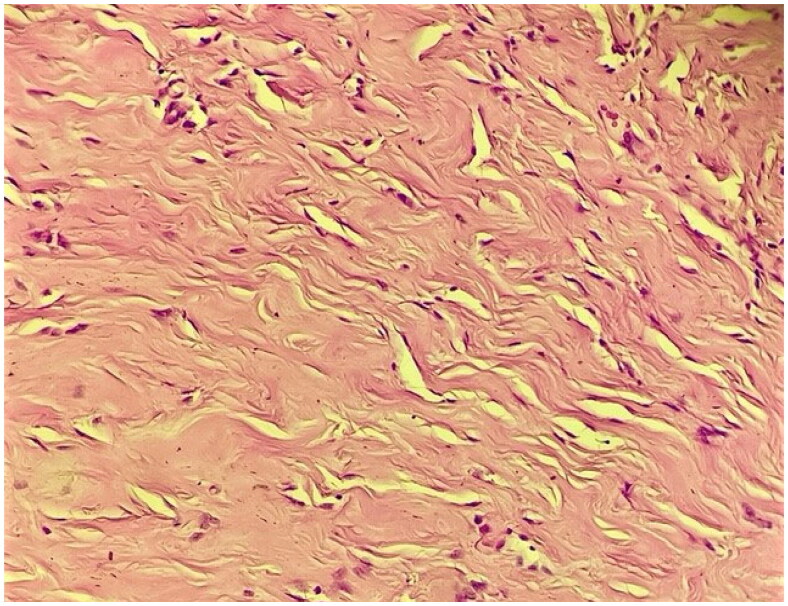
High power view showing pseudoangiomatous stromal hyperplasia, with multiple slit-like spaces often displaying myofibroblasts at their margins that simulate endothelial cells (40× Magnification; Hematoxylin-Eosin staining).

**Figure 3.**
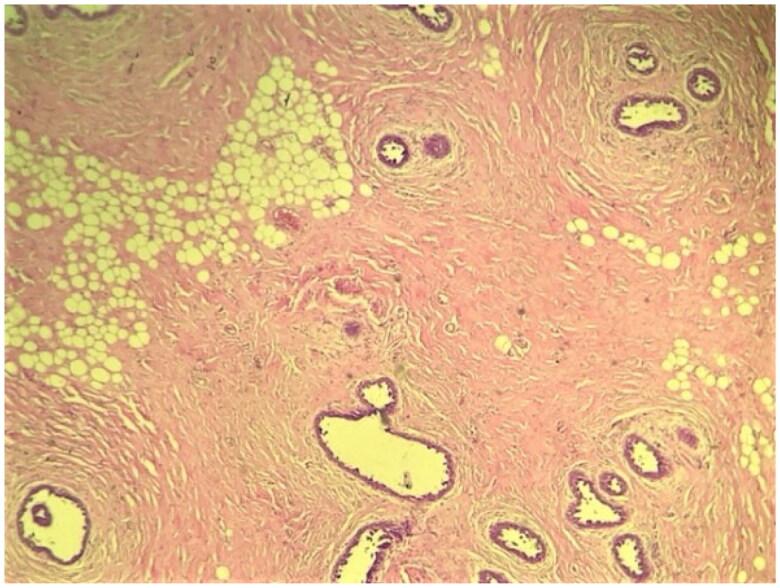
Low power view showing breast parenchyma displaying an increased number of ducts with a stromal proliferation (4× Magnification; Hematoxylin-Eosin staining).

**Figure 4.**
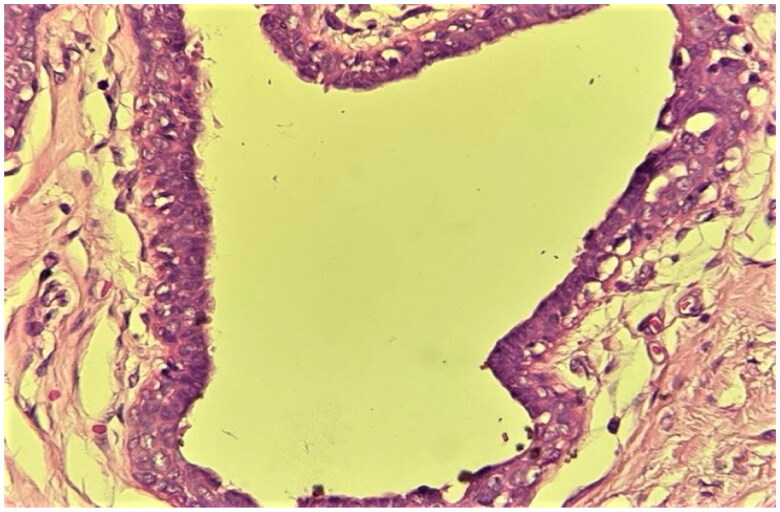
High power view showing a duct lined by a three-layer epithelium (luminal, intermediate and myoepithelial), cuffed by a loose stroma. (40× Magnification; Hematoxylin-Eosin staining).

The patient underwent surgery, which consisted of the removal of the male breast tissue through an infra-areolar incision, whilst preserving the overlying nipple areola complex. Histological finding resulted in a mammary fibrous stroma containing ductal hyperplasia. The ducts are present in a dense hyalinized stroma which shows features of pseudoangiomatous stromal hyperplasia (Figures 2–4).

The postoperative follow-up showed no complications. The patient was stable, and satisfied with the aesthetic outcome. However, 8 months post-operatively, the patient developed a mild enlargement of the nipple-areola complex and was candidate for a secondary glandular resection, but he refused to undergo another operation due to satisfaction with current results.

## Discussion

Enlargement of the male breast is caused by gynecomastia in more than 80% of cases, 75% of them are due to a hormonal imbalance: an increase in estradiol concentration, lagging free testosterone production, and increased tissue sensitivity to normal male levels of estrogen. Estradiol acts as a growth hormone of the breast and therefore an excess of estradiol in men leads to the proliferation of breast tissue [4].

The microscopical appearance of gynecomastia depends on the duration of the disease; at the early stage of gynecomastia formation, the stroma is rather cellular, edematous, and commonly shows pseudo angiomatous stromal hyperplasia (PASH). Later, the stroma becomes progressively dense and fibrous [4].

This further clarifies the relationship between pseudoangiomatous hyperplasia and the initial and intermediate phases of gynecomastia where epithelial proliferation is predominant as reported by Ibrahim and al [5] and Badve and Sloane [3].

Pseudoangiomatous stromal hyperplasia (PASH) is a benign proliferative lesion of the mammary stroma containing complex anastomosing spaces that may be confused with angiosarcoma on histological analysis. It is very similar histologically to the normal mammary stroma during the luteal phase of the menstrual cycle [1]. Therefore, it has been suggested that PASH may represent an over stimulation by progesterone on estrogen-primed breast stromal tissue.

Although considered a rare entity, with an incidence that is difficult to estimate given the paucity of reports [5], PASH is most commonly found incidentally in pre-menopausal women with an age range from 14 to 67 years old [6], though some rare infant cases have been reported [7]. It may clinically present as a nodule and mimic a fibroadenoma [7], or rarely as a rapidly growing bilateral mass [8], which represents another entity which is tumoral PASH [9]. Some cases in ectopic mammary-like glands have also been described [10,11].

In men, according to literature, there have been eight reported cases of PASH affecting male patients published to date [12]. This case features the 9th known PASH case in males. It was firstly discovered in men by Badve and Sloane in 1995 [3], 93 consecutive male breast specimens were reviewed which led to the identification of 47.4% cases showing at least one focus of pseudoangiomatous hyperplasia. All were associated (both clinically and histologically) with gynecomastia.

These findings were confirmed by Milanezi et al. in 1998 [13]. Their study showed that in 88 cases of gynecomastia, 21 of them (22%) were diagnosed with pseudoangiomatous hyperplasia of mammary stroma. Even more so when affecting males.

PASH is often diagnosed during a core needle biopsy in pre and perimenopausal women, its management when histologically confirmed, doesn’t require a specific treatment, a close observation with serial mammography seems to be adequate treatment [14,15]. Progression of the lesion however, often prompts simple excision with clear margins to prevent recurrences [16]. Whereas, rapidly growing masses might require mastectomy. A systemic treatment with tamoxifen has been reported to be effective in a young female patient presenting a breast enlargment, pain and breast masses [17]. But further studies will be needed to establish it as a definitive medical treatment.

In our case, the patient developed a recurrent gynecomastia 8 months after the first excision even after a wide mastectomy leaving a thin glandular tissue behind the nipple areola complex to avoid the areola to shrink. The incidence of gynecomastia recurrence following plastic surgery treatment remains elusive, owing largely to a variety of techniques used, varying degrees of gynecomastia [18]. Nonetheless, authors of [19] identified that even with the most aggressive approaches, such as mastectomies, glandular ­gynecomastia recurrence was still noted in 12.5% of patients [19].

In the other hand, in literature, PASH is not frequent in gynecomastia specimen, and its recurrence is even less described. The recurrence rates in women in the pathology literature range from 15% to 22% [9]. This phenomenon could be attributable to growth of a residual mass after incomplete excision, the presence of multiple lesions that were not all excised, or de novo growth of PASH.

When found in gynecomastia specimens, no additional treatment is required since PASH is considered to be a benign lesion that can be found in normal male breast tissue. A follow-up, however, is advised.

## Conclusion

Pseudoangiomatous stromal hyperplasia (PASH), although primarily described in women, is commonly observed in young men with confirmed gynecomastia. It has been established that PASH is linked to the initial phases of mammary stroma maturation. Importantly, PASH is not a precancerous condition and generally carries a favorable prognosis. Consequently, the identification of this benign lesion does not alter the treatment approach or prognosis for gynecomastia.
